# Labeling Proteins Inside Living Cells Using External Fluorophores for Fluorescence Microscopy

**DOI:** 10.7554/eLife.25460

**Published:** 2017-01-31

**Authors:** Kai Wen Teng, Yuji Ishitsuka, Pin Ren, Yeoan Youn, Xiang Deng, Pinghua Ge, Sang Hak Lee, Andrew S Belmont, Paul R Selvin

Teng KW, Ishitsuka Y, Ren P, Youn Y, Deng X, Ge P, Belmont AS, Selvin PR. 2016. Labeling proteins inside living cells using external fluorophores for microscopy. *eLife* **5**:e20378. doi: 10.7554/eLife.20378.

Published 9, December 2016

Dr. Sang Hak Lee has been left out as a co-author accidentally. His contribution to fiduciary patterned surface with unique address that was utilized in figure 1-figure supplement 1b and c has been overlooked due to miscommunication. The corrected list of authors and their affiliations is below:

Kai Wen Teng^1,2^, Yuji Ishitsuka^2,3^, Pin Ren^1,2^, Yeoan Youn^1,2^, Xiang Deng^4^, Pinghua Ge^3^, Sang Hak Lee^2,3^, Andrew S Belmont^1,4^, Paul R Selvin^1,2,3,4*^

^1^Center for Biophysics and Quantitative Biology, University of Illinois at Urbana-Champaign, Urbana, United States; ^2^Center for Physics of Living Cell, University of Illinois at Urbana-Champaign, Urbana, United States; ^3^Department of Physics, University of Illinois at Urbana-Champaign, Urbana, United States; ^4^Department of Cell and Developmental Biology, University of Illinois at Urbana-Champaign, Urbana, United States

Additional information for Dr. Sang Hak Lee:

**Funding:**

National Science Foundation NSF PHY-143124

National Institutes of Health NIH GM 108578

**Author Contribution:** SL, Conception and design.

Second, we have left out an important step that leads to activation of SLO in our methods section under the heading of “Reversible permeabilization and probe loading”. The additional sentence should read “An aliquot of SLO stock was reduced by 10 mM TCEP (Pierce) for 20 minutes at 37 oC.” and is to be inserted between “A new aliquot was thawed prior to each experiment and used within 5 hr.” and “The SLO stock solution was further diluted to working concentration in DPBS supplemented with 1 mM MgCl2.”. The sentence was present in previous edition of the draft, but was left out due to mistake in editing. We thank the reader for pointing this out to us.

The article has been corrected accordingly.

